# Populations of Select Cultured and Uncultured Bacteria in the Rumen of Sheep and the Effect of Diets and Ruminal Fractions

**DOI:** 10.1155/2011/750613

**Published:** 2011-07-21

**Authors:** Jill Stiverson, Mark Morrison, Zhongtang Yu

**Affiliations:** ^1^Department of Animal Sciences, The Ohio State University, Columbus, OH 43210, USA; ^2^CSIRO Livestock Industries, St. Lucia, QLD 4607, Australia

## Abstract

The objective of this study was to assess the importance of select cultured and uncultured bacteria in the rumen by quantifying their populations and the effect of diets and ruminal fractions. Full-length 16S rRNA gene (*rrs*) sequences were recovered from rumen samples using specific primers designed from partial sequences recovered previously. Five uncultured bacterial operational taxonomic units (OTUs) were quantified using specific quantitative PCR (qPCR) in fractionated ruminal samples from sheep fed either hay alone or hay plus corn. Species *Fibrobacter succinogenes*, *Ruminococcus albus*, *R. flavefaciens*, *Ruminobacter amylophilus*, *Selenomonas ruminantium*, and *Mitsuokella multacida* and genera *Butyrivibrio* and *Prevotella* were also quantified as comparison. The full-length *rrs* sequence improved taxonomic assignments of partial *rrs* sequences. Genus *Prevotella* had the greatest abundance. Of the three major cultured cellulolytic species, *R. flavefaciens* was most abundant, followed by *R. albus* and *F. succinogenes*. The five uncultured bacterial OTUs, classified to genus *Acetivibrio*, genus *Allobaculum*, family *Ruminococcaceae*, order *Clostridiales*, or class *Clostridia*, had abundance comparable to that of the above species of genera except *Prevotella*. Corn supplementation and fractions affected distribution of the rumen bacteria, but to a limited extent. When compared to the qPCR data, sequence frequencies in the *rrs* clone libraries tended to overestimate the abundance of the bacteria represented. This study showed that abundance and population dynamics of uncultured bacteria can be quantified by specific qPCR, which complements the results of *rrs* clone libraries. This study also revealed that some uncultured bacteria might be as important as some of the well-characterized bacteria in the rumen. The approach used should be applicable to assess the abundance and potential importance of uncultured bacteria in other environments.

## 1. Introduction

The complex rumen microbiome plays essential roles in digesting feeds and supplying nutrients to host animals. To positively affect rumen functions, dietary interventions have been attempted to modulate this microbiome [1]. The rumen microbiome as a whole and its individual populations are often analyzed to unveil the underpinning of dietary interventions. Community fingerprinting by DGGE [2], T-RFLP [3], and ARISA [4] have been used in accessing overall dietary effects. However, detailed assessments come from analysis of 16S rRNA gene (*rrs*) clone libraries or quantitative comparisons of populations of known species or groups, such as *F. succinogenes*, *R. albus*, *R*. *flavefaciens*, *Butyrivibrio fibrisolvens* [5, 6], *Prevotella *spp. [7], *Megasphaera elsdenii * [8], and methanogens [9]. However, most of these known species or populations account for only a small portion of the rumen microbiome, as exemplified by *F*. *succinogenes*, *R*. *albus*, and *R*. *flavefaciens*, which together represent only 4.5–9% of the total bacteria in the rumen of sheep [10]. 

Analysis of *rrs* clone libraries can identify both known (i.e., cultured) and novel bacteria (i.e., uncultured). Of the bacteria identified by *rrs* sequences of rumen origin, cultured bacteria only accounted for 6.5% [11]. Large numbers of uncultured bacteria represented by novel *rrs* sequences were especially found within families *Ruminococcaceae*, *Lachnospiraceae*, and order *Clostridiales*. Function and ecology of uncultured bacteria are often inferred from closely related species, while their relative abundance, and thus weight or importance to the entire microbiome, is typically estimated from prevalence of respective *rrs* sequences in clone libraries. However, sequence prevalence does not necessarily reflect actual abundance of the represented bacteria in the microbiome because of biases associated with PCR and cloning [12]. We hypothesize that the population sizes of uncultured bacteria can be quantified using specific qPCR, and the quantitative information can complement the results of *rrs* clone libraries and help gauge the importance of uncultured bacteria. In this study, we tested this hypothesis by recovering full-length *rrs* sequences from select uncultured bacteria and then quantifying their abundance in fractionated rumen samples of sheep fed hay only or hay plus corn. The populations of select well-characterized ruminal bacteria were also quantified for comparison.

## 2. Materials and Methods

### 2.1. Feeding, Sample Collection, and DNA Extraction

Four ruminally cannulated sheep were divided into two groups of two sheep each and used in the feeding experiment that was set up in a crossover design with two periods of three weeks each. During the first period, one group was fed 100% orchardgrass hay, while the other group was fed a combination of 70% orchardgrass hay and 30% corn. During the second period, the diets were switched. The sheep were fed once daily for 21 days prior to ruminal sample collection, which took place 6 hrs after feeding. Sample collection and fractionation (adhering or Ad versus liquid or Lq) were done as described previously [13]. Metagenomic DNA was extracted using the RBB+C method [14]. The DNA quality was evaluated using agarose gel (1.0%) electrophoresis, and DNA yield was quantified using the Quant-it Kit (Invitrogen Corporation, Carlsbad, Calif, USA). 

### 2.2. Cloning, Sequencing, and Phylogenetic Analysis

**Figure 1:**
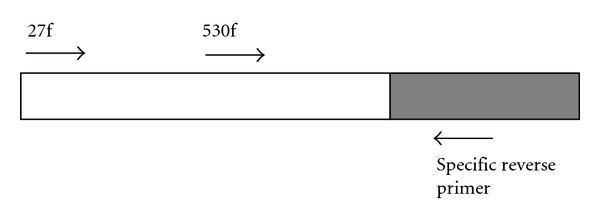
A diagram showing the position and the approach used to obtain full-length *rrs* gene. The shaded box represents the downstream partial sequence determined in a previous study [13], while the open box represents the upstream partial sequence determined in this study. Each sequence-specific reverse primer was used to retrieve the upstream partial sequence and to quantify the abundance of each of the uncultured bacteria when paired with bacterial primers 27f and 5330f, respectively.

In a previous study [13], a number of OTUs were defined from novel partial *rrs* sequences (approximat 600 bp from the 3′ end corresponding to 900–1,540 of the *E. coli rrs* gene) recovered from fractionated rumen samples. Some of these downstream partial sequences either have no close match in the RDP database or only match sequences of bacteria never reported in the rumen. One specific reverse primer was designed using Primer Premier 5.0 (Premier Biosoft Int'l, Palo Alto, Calif, USA) for each of 25 OTUs defined from the downstream partial sequences. The specificity of the primers was verified by *in silico* analysis against sequences in the RDP database using the Probe Match function. This OTU-specific reverse primer (Table 1) was paired with bacterial primer 27f (Table 2) in PCR to recover the upstream partial sequence. The positions of the primers were shown in Figure 1. The PCR reactions and cloning were done as described previously [13], except for an optimized annealing temperature at 54°C. Three random clones were chosen from each clone library for sequencing from both ends. The partial sequences recovered from each clone library were assembled using BioEdit [15] to form the upstream partial sequence (>1000 bp from the 5′ end corresponding to 8–1006 or beyond of the *E. coli rrs* gene). This upstream partial sequence and the corresponding downstream partial sequence recovered previously [13] were assembled into a full-length gene sequence using BioEdit [15]. The overlap between the two partial sequences ranged from 106 to 566 bp. The full-length sequences were subjected to vigorous chimera analysis using four different programs: the RDP Chimera Check (http://rdp.cme.msu.edu/), the Bellerophon (http://greengenes.lbl.gov/), the Pintail program of Bioinformatics-Toolkit (http://www.bioinformatics-toolkit.org/Web-Pintail/), and the Mallard [16]. All suspect chimeric sequences were excluded from further analysis. The full-length sequences were compared to RDP sequences and classified using the Classifier program [17]. A neighbor-joining tree based on the full-length sequences and their most similar sequences was constructed using the neighbor Joining method at RDP [18]. The full-length sequences obtained in this study were deposited in GenBank (accession numbers GU120110, GU120113, GU120120, GU120121, GU120128, GU120129, and GU120131-GU120137).

### 2.3. Quantification of Cultured Bacteria and Uncultured Bacteria by Specific qPCR

One qPCR standard was prepared for each species, genus, group, or OTU of the uncultured bacteria to be quantified. The standard for *F. succinogenes*, *R. albus*, and *Prevotella* was prepared through PCR using the bacterial primers 27f and 1525r (Table 2) and the genomic DNA of *F. succinogenes* S85, *R. albus* 8, and *Prevotella ruminicola* 23, respectively. Due to the lack of strains in our laboratory, a sample-derived standard was prepared for genus *Butyrivibrio*, *R. amylophilus*,* R. flavefaciens*, and *S. ruminantium*, total bacteria, and total archaea using the respective specific PCR primer set (Table 2) and a composite DNA sample pooled from all the metagenomic DNA to be quantified as described previously [25]. One sample-derived standard for each of the uncultured bacterial OTUs was prepared similarly but with PCR using primer 530f (Table 2) and the respective specific reverse primer for each of the full-length *rrs* sequences (Table 1). The standards were purified and quantified as done previously [25]. Each standard was serially diluted (1 : 10) immediately before the qPCR assays, and the concentrations ranged from 10^1^ to 10^7^ copies per reaction. 

qPCR was carried out using respective specific primers (Table 2) and a Stratagene Mx3000 machine (La Jolla, Calif, USA). Total bacteria was quantified using the TaqMan assay [20], while the other species, groups or uncultured bacterial OTUs were quantified using SYBR Green-based qPCR. The PCR conditions were the same as those used previously [25], except for the annealing temperature that was optimized in this study (see Table 2). Each of the uncultured bacterial OTUs was quantified using the respective OTU-specific reverse primer (Table 1) and bacterial primer 530f (Table 2) similarly as for the cultured bacteria but with a primer annealing temperature at 54°C. To minimize variations, the qPCR assay for each bacterium, species, or group was done in triplicates for both the standards and the metagenomic DNA samples using the same master mix and the same PCR plate. No-template controls were included in triplicates in parallel. 

Absolute abundance was calculated as *rrs* copied per *μ*g metagenomic DNA, while relative abundance was expressed as percent of total bacterial *rrs* copies. The absolute abundance was not expressed as *rrs *gene copies/g or mL of sample because (i) the solid and the liquid fractions have different sample matrices, (ii) not all adhering bacteria can be detached or recovered from the solid digesta particles [13], and (iii) the two types of diets probably resulted in solid digesta particles containing different contents of plant content materials (nonmicrobial). The mean was calculated from the three replicates of the qPCR for each sample. Then, the mean was calculated from the four samples (two animals by two periods) of each fraction and each diet. The fraction- and diet-based data of abundance were analyzed using one-way analysis of variance (ANOVA) using GraphPad Prism 5 (GraphPad Software, San Diego, Calif, USA) and the means were compared using one-way ANOVA with Tukey's multiple comparison test. Significant difference was declared at *P* ≤ 0.05, while tendency was declared at *P* ≤ 0.10.

## 3. Results

### 3.1. Phylogenetic Assignment of the Full-Length rrs Sequences

**Figure 2:**
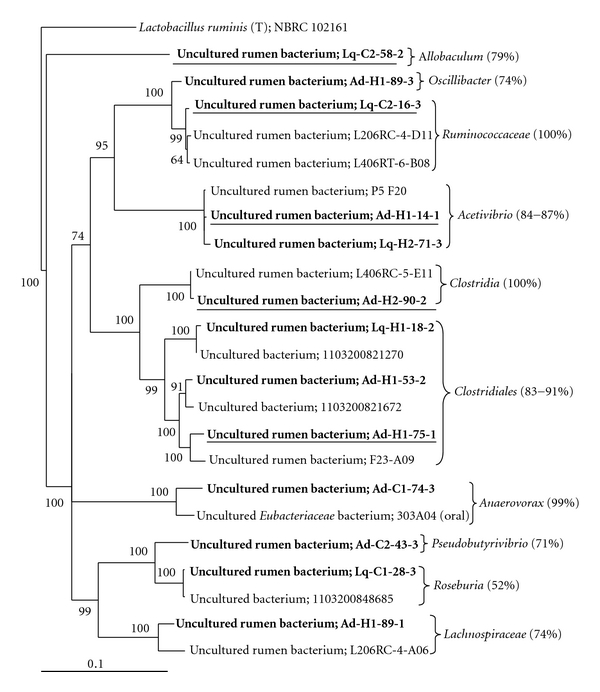
A Weighbor-joining tree based on the full-length sequences recovered in this study and their mostly similar sequences found in the RDP database. The taxa to which the sequences were assigned to are also shown. The values in brackets are the confidence level obtained from the Classifier program of RDP. The tree was rooted with the sequence of *Lactobacillus ruminis* (T); NBRC 102161. The numbers at branching points are bootstrap values based on 100 replicates. The scale bar represents 0.1 substitution per site. Sequences obtained in this study were shown in bold. The uncultured bacteria that were quantified by qPCR were also underlined.

Twenty-five reverse primers were designed and used in retrieving upstream partial sequences, but only 12 each were allowed for identical upstream partial sequences from the clones that were sequenced. The remaining 13 primers generated 2-3 very similar but different upstream partial sequences (data not shown). These latter primers and sequences were excluded from further analysis. The full-length sequences were assigned to genus (the lowest taxonomic rank of the new Bergey's Taxonomy of Prokaryotes used in the RDP database) or higher taxa (Figure 2). The increased sequence length allowed taxonomic assignment with a greater confidence for all the sequences and to genus for Ad-H1-89-3, Ad-C2-43-3, Ad-C1-74-3, Lq-C1-28-3, and Lq-C2-58-2. Six full-length sequences remained to be assigned to a genus (i.e., Lq-C2-16-3 and Ad-H2-89-1), family (i.e., Lq-H1-18-2, Ad-H1-53-2, and Ad-H1-75-1), or order (i.e., Ad-H2-90-2) (Figure 2). Seven of the full-length sequences each are very similar (99% sequence identity) to sequences also recovered from the rumen by other researchers (based on BLASTn analysis and GenBank records), including Lq-H1-18-2 (17 sequences), Lq-H2-71-3 (9 sequences), Ad-H1-14-1 (45 sequences), Lq-C1-28-3 (7 sequences), Lq-C2-16-3 (2 sequences), Ad-H1-53-2 (1 sequence), and Ad-H2-90-2 (1 sequence). Because none of these novel bacteria can be classified to existing species or genus, they were referred to bacterial OTUs. These OTUs, especially OTUs Ad-H1-14-1 and Lq-H1-18-2, may represent bacteria common in the rumen. The remaining six full-length sequences shared no more than 97% sequence identity with any of the sequences in the RDP or the GenBank. These sequences likely represent novel bacteria that have not been documented. 

### 3.2. Quantification of Cultured Bacteria

 Total bacteria did not differ in abundance between the two diets or between the two fractions, while the archaeal abundance tended to be lower (*P* = 0.067) in the H-Ad sample than in the other three samples (Table 3). Of the three major known cellulolytic species, *R. albus* and* R. flavefaciens *were more abundant than *F. succinogenes* in all the samples (Table 3), a finding consistent with some previous studies (e.g., [26, 27]) but contradictory to other studies (e.g., [28, 29]). *F. succinogenes *tended to be more abundant in the adhering fraction and in the hay-fed sheep, reflecting its ability to adhere to fiber particles in the rumen. The abundance of the two *Ruminococcus* species, especially relative to total bacteria, was numerically higher in the adhering fraction of hay-fed sheep. Overall, the abundance of each of these three cellulolytic species was similar between the two diets and between the two fractions. Genus *Butyrivibrio* was significantly more abundant in the hay-fed sheep than in the hay:corn-fed counterparts, but its abundance did not differ between the two ruminal fractions within the same dietary group (Table 3). Of the known species and genera quantified, genus *Prevotella* was the most predominant, accounting for about 27% of the total bacteria in the hay-fed sheep and more than 50% in the hay:corn-fed sheep (Table 3). *R. amylophilus* was found in relatively high abundance in the adherent fraction of the sheep fed the hay:corn diet, corresponding to its ability to utilize starch. *S. ruminantium *and *M. multacida *are closely related species within family *Veillonellaceae*. Since the primers used in this study amplify both species, they were quantified together as a group. As shown in Table 3, this group of bacteria was less abundant in the adhering fraction of the hay-fed sheep than in the liquid fraction of the hay:corn-fed sheep. These results are in general agreement with the ability of these two species to utilize sugars and lactate. 

### 3.3. Quantification of Uncultured Bacteria

The abundance of OTUs Ad-H1-14-1 (classified to genus *Acetivibrio*), Lq-C2-16-3 (classified to *Ruminococcaceae*), and Ad-H2-90-2 (classified only to class *Clostridia*) ranged from 10^6^ to 10^7^ copies of *rrs* genes/*μ*g metagenomic DNA, corresponding to about 0.11 to 1.2% of total bacteria (Table 3). These abundances were lower than that of the genus *Prevotella*, comparable to that of the two *Ruminococcus* species and the genus *Butyrivibrio,* but higher than that of *F. succinogenes*, *R. amylophilus, *or *S. ruminantium *and *M. multacida *combined. These three OTUs did not differ in abundance between the two fractions in the hay-fed sheep, but appeared to be numerically more abundant in the adhering fraction than in the liquid fraction of the hay:corn-fed sheep. OTUs Lq-C2-58-2 (classified to genus *Alloculum*) and Ad-H1-75-1 (classified only to order *Clostridiales*) were slightly less abundant than the other three OTUs mentioned above. OTU Lq-C2-58-2 was significantly more abundant in the adhering fraction than in the liquid fraction of the hay:corn-fed sheep and tended to be more abundant in the liquid fraction of hay-fed sheep. Overall, OTUs Ad-H1-14-1, Ad-H2-90-2, and Ad-H1-75-1 were more abundant in the hay-fed sheep than in the hay:corn-fed sheep, whereas OTUs Lq-C2-16-3 and Lq-C2-58-2 had comparable abundance between the two feeding groups.

## 4. Discussion

Due to cost and time restraint, partial *rrs* gene sequences are typically determined in most studies that examined the diversity and species richness of various microbiome samples. As demonstrated in this study, however, full-length *rrs* sequences improve upon partial sequences in classification of *rrs* sequences, in both confidence and low taxa that are more informative. Although this is expected, the results of this study demonstrated the extent and aspects that full-length *rrs *sequences can improve over partial *rrs* sequences. Conceivably, full-length high-quality *rrs* sequences are also important in assigning candidate taxa (*incertae sedis*) that do not have cultured representatives. It should be noted that 13 of the 25 reverse primers designed in this study did not allow for retrieval of a single upstream partial sequence. Given that these reverse primers did not have any match in RDP or GenBank (data not shown), this might be explained by the fact that the downstream regions (V6–V9) of the *rrs* genes are more conserved than the upstream regions [30, 31]. 

All the full-length sequences determined in this study represented uncultured ruminal bacterial OTUs that cannot assigned to existing species or genera. Classification to a genus helped with inference of possible functions of these bacterial OTUs, such as OTUs Ad-H1-14-1 (classified to *Acetivibrio*), Ad-C1-74-3 (classified to *Anaerovorax*), and Lq-C1-28-3 (*Roseburia*). In addition to 45 nearly identical sequences, OTUs Ad-H1-14-1 is 98.8% identical to the sequence of a new strain, R-25 (accession number of its *rrs* sequence: AB239489), which was recently isolated from an enriched culture of sheep rumen [32]. This strain was shown to be short rod or coccus shaped, to adhere to orchardgrass hay, and to have activities of carboxymethylcellulase, xylanase, and *α*-L-arabinofuranosidase. Strain R-25 reached a relative abundances of 0.98% and 0.64% of total bacteria in the solid and the liquid fractions of rumen samples collected from sheep [32], which is comparable to that of OTU Ad-H1-14-1 in the sheep rumen sampled in this present study (Table 3). *Acetivibrio *is a genus that contains only two recognized cellulolytic species (i.e., *A. cellulolyticus* and *A*. *cellulosolvens*). This genus has no cultured representative from the rumen. However, the high prevalence of OTU Ad-H1-14-1 sequences recovered from the rumen and the relatively high abundance of Ad-H1-14-1 determined in the hay-fed sheep than in the hay:corn-fed sheep (Table 3) suggest that OTU Ad-H1-14-1, together with strain R-25, may represent an important species of fibrolytic ruminal bacteria in the genus *Acetivibrio*. 

In this study, we quantified the populations of five uncultured bacteria classified to known genera (i.e., OTUs Lq-C2-58-2 and Ad-H1-14-1), family (i.e., OTU Lq-C2-16-3), order (OTU Ad-H1-75-1), or class (OTU Ad-H2-90-2) in the phylum *Firmicutes* (Figure 2). These uncultured bacteria had abundance comparable to or greater than that of known species or genera (except the genus *Prevotella*) of bacteria that are perceived important to rumen functions. For example, OTU Ad-H1-14-1 may be one important group of bacteria participating in fiber digestion in the rumen. Additionally, OTU Lq-C2-58-2 was classified to genus *Allobaculum *within the class *Erysipelotrichi*, a class of *Firmicutes *poorly represented by sequences of rumen origin [11]. *Alloculum* is a new genus represented by a single species, *A. stercoricanis*, whose type strain was initially isolated from canine feces and was shown to utilize several sugars, including glucose, fructose, maltose, and cellubiose [33]. OTU Lq-C2-58-2 might represent a group of scavenger utilizing sugars in the rumen. Although the specific functions of these uncultured bacteria remain to be determined, their relatedness with known taxa and their abundance suggest that some of them may play as important role in rumen function as some of the well-characterized bacteria. 

The abundance of a bacterial group in an environment reflects its ability to adapt to the conditions and compete for the nutrients available therein and signifies its importance to the overall functions of the microbiome. As mentioned above, sequence frequencies in *rrs* gene clone libraries are often used in assessing the relative abundance of the bacteria represented, but the validity of such correlation is uncertain due to bias inherent to PCR and cloning [12]. In this study, we examined the above validity by comparing the frequency of five downstream partial sequences that were recovered previously in clone libraries [13] and the relative abundance (% of total bacteria) of the corresponding full-length sequences quantified by the specific qPCR assays. In the clone libraries, sequence Ad-H1-75-1 had a frequency of 2.1%, while the other four sequences had a frequency of 1.0%, in the fractions from which these sequences were recovered. The frequencies of all these sequences were greater than their relative abundance determined in this study, especially Ad-H1-75-1 (Table 3), suggesting that these sequences were over represented in the *rrs* gene clone libraries and their frequency in the clone libraries would overestimate their abundance in the rumen. More studies involving multiple *rrs* gene clone libraries constructed using different PCR primers and from different samples are needed to verify if sequence frequency in *rrs* gene clone libraries generally overestimates relative abundance of the bacteria represented. Nevertheless, caution should be exercised in estimating bacterial abundance from sequence frequency in *rrs* gene clone libraries. As demonstrated in this study, actual abundance and population dynamics of uncultured bacteria should be quantified using specific qPCR so their ecology and importance to the microbiome can be inferred more accurately.

## Figures and Tables

**Table 1:** Primers* used to retrieve full-length *rrs* gene sequences of the uncultured bacteria.

Partial sequences (GenBank accession no.)	Sequences (5′ → 3′)	Annealing position (*E. coli* numbering)	Amplicon length (bp)
Lq-H1-18 (AY816400)	GAC ACA CCT GAT CTC TCA GGT T	1006–1028	1,021
Ad-H1-89 (AY816504)	CGA CTT TGC TTC CCT CTG TTT	1246–1266	1,259
Ad-C2-43 (AY816609)	CTC CAG AGT GCC CAT CCG AA	1135–1154	1,147
Ad-C1-74 (AY816616)	GAA GGG ACC GGT TAA GGT C	1013–1031	1,024
Lq-H2-71 (AY816389)	TTC TCG GCC CCA AAT TCG	1466–1483	1,476
Ad-H1-14 (AY816508)	GAT TTG CTT ACC CTC GCG GGT TT	1260–1282	1,275
Ad-H1-53 (AY816423)	CGT ATC TCT ACG GCT TTA C	1002–1020	1,013
Ad-H1-75 (AY816420)	CAC ACC TTG TAT CTC TAC AAG C	1006–1027	1,020
Ad-H2-90 (AY816432)	CTT CGA CAG CTG CCT CCT TA	1451–1470	1,463
Lq-C1-28 (AY816538)	CCA GGG CCA TTA CAC CCT GT	1005–1025	1,018
Lq-C2-16 (AY816578)	GAC TTT GCT TCC CTT TGT TTT G	1245–1265	1,258
Lq-C2-58 (AY816550)	AGC CTC CGA TAC ATC TCT GC	1010–1029	1,022

*Only the primers that produced a single sequence were listed.

**Table 2:** Primers and probe used for real-time PCR quantification of recognized rumen bacterial species, genera, or uncultured bacteria.

Primers	Sequences (5′ → 3′)	Target	Annealing temperature (°C)	Amplicon length (bp)	References
27f	AGA GTT TGA TCM TGG CTC AG	Bacteria	54	1,535	[19]
1525r	AAG GAG GTG WTC CAR CC				
340f	TCC TAC GGG AGG CAG CAG T	Bacteria	60	467	[20]
806r	GGA CTA CCA GGG TAT CTA ATC CTG TT				
TaqMan probe	6-FAM-5′-CGT ATT ACC GCG GCT		70		
	GCT GGC AC-3′-TAMRA				
Bac303f	GAA GGT CCC CCA CAT TG	*Bacteroides *and *Prevotella *	56	418	[21]
Bac708r	CAA TCG GAG TTC TTC GTG				
ARC787F	ATT AGA TAC CCS BGT AGT CC	Archaea	60	273	[22]
ARC1059R	GCC ATG CAC CWC CTC T				
Ra1281f	CCC TAA AAG CAG TCT TAG TTC G	*R. albus*	55	175	[23]
Ra1439r	CCT CCT TGC GGT TAG AAC A				
Fs-f	GGT ATG GGA TGA GCT TGC	*F. succinogenes*	63	446	[24]
Fs-r	GCC TGC CCC TGA ACT ATC				
530f	GTG CCA GCM GCC GCG G	*Butyrivibrio*	65	371	[19] and this study
Buty-900r	TGC GGC ACY GAC TCC CTA TG				
Sel-Mit-f	TGC TAA TAC CGA ATG TTG	*S. ruminantium *and *M. multacida *	53	513	[24]
Sel-Mit-r	TCC TGC ACT CAA GAA AGA				
Ram-f	CAA CCA GTC GCA TTC AGA	*Ruminobacter amylophilus*	57	642	[24]
Ram-r	CAC TAC TCA TGG CAA CAT				
Rf154f-K	TCT GGA AAC GGA TGG TA	*R. flavefaciens*	55	295	[23]
Rf425r-K	CCT TTA AGA CAG GAG TTT ACA A				

**Table 3:** Populations of bacteria (cultured and uncultured) and archaea.

	H-Ad	H-Lq	C-Ad	C-Lq	SEM	*P* value
Total bacteria	3.6×10^9^	8.3×10^9^	5.3×10^9^	4.8×10^9^	1.0×10^9^	0.123
*F. succinogenes*	2.7×10^5 (a)^ (0.01%)	5.6×10^4 (a,b)^ (0.00%)	1.3×10^5 (a,b)^ (0.00%)	2.1×10^4 (b)^(0.00%)	5.6×10^4^ (0.001%)	0.025 (0.004)
*R. albus*	6.5×10^8^ (17.94%)	1.5×10^7^ (0.18%)	4.9×10^7^ (0.94%)	3.1×10^7^ (0.65%)	1.5×10^8^ (0.43%)	0.477 (0.398)
*R. flavefaciens*	5.9×10^7^ (1.63%)	4.3×10^7^ (0.52%)	5.3×10^7^ (1.01%)	5.0×10^7^(1.05%)	3.2×10^6^ (0.23%)	0.984 (0.307)
*Butyrivibrio*	8.7×10^7 (a)^ (2.42%)	8.7×10^7 (a)^ (1.05%)	6.3×10^6 (b)^ (0.12%)	2.5×10^6 (b)^ (0.05%)	2.4×10^7^ (0.55%)	0.003 (0.001)
*Prevotella*	9.7×10^8^ (26.97%)	2.3×10^9^ (26.95%)	2.8×10^9^ (53.65%)	2.9×10^9^ (60.67%)	4.5×10^8^ (8.83%)	0.459 (0.255)
*R. amylophilus*	8.6×10^3^ (0.00%)	1.6×10^4^ (0.00%)	8.2×10^4^ (0.00%)	1.0×10^4^(0.00%)	1.8×10^4^ (0.00%)	0.131 (0.231)
*S. ruminantium *&* M. multacida *	2.8×10^6 (a)^ (0.08%)	4.7×10^7 (a,b)^ (0.56%)	6.1×10^7 (a,b)^ (1.15%)	1.5×10^7 (b)^ (0.30%)	1.4×10^7^ (0.23%)	0.051 (0.060)
Ad-H1-75-1	5.0×10^6^ (0.14%)	9.2×10^6^ (0.11%)	2.3×10^6^ (0.04%)	3.4×10^6^ (0.07%)	1.5×10^6^ (0.02%)	0.072 (0.334)
Ad-H2-90-2	1.2×10^7 (a)^ (0.33%)	4.6×10^7 (b)^ (0.56%)	1.0×10^7 (a)^ (0.19%)	5.4×10^6 (a)^ (0.11%)	9.4×10^6^ (0.10%)	0.007 (0.196)
Ad-H1-14-1	4.5×10^7^ (1.24%)	4.5×10^7^ (0.54%)	1.3×10^7^ (0.25%)	9.9×10^6^ (0.21%)	9.6×10^6^ (0.24%)	0.327 (0.095)
Lq-C2-16-3	2.5×10^7^ (0.68%)	2.4×10^7^ (0.28%)	4.9×10^7^ (0.94%)	2.7×10^7^ (0.57%)	6.1×10^6^ (0.14%)	0.518 (0.320)
Lq-C2-58-2	8.9×10^6^ (0.25%)	1.9×10^7^ (0.23%)	2.9×10^7^ (0.55%)	4.6×10^6^ (0.10%)	5.5×10^6^ (0.10%)	0.095 (0.094)
Methanogens	4.0×10^5^	2.5×10^6^	1.7×10^6^	2.4×10^6^	4.9×10^5^	0.068

Note: Means within a row with different superscripts differ (*P* < 0.05). Values in parentheses are relative abundance (%) of total bacteria.
